# Virtual reality-assisted cognitive behavioral therapy for patients with alcohol use disorder: a randomized feasibility study

**DOI:** 10.3389/fpsyt.2024.1337898

**Published:** 2024-02-14

**Authors:** Daniel Thaysen-Petersen, Sigurd Krogh Hammerum, Anne-Cathrine Vissing, Irene Henriette Oestrich, Merete Nordentoft, Signe Wegmann Düring, Anders Fink-Jensen

**Affiliations:** ^1^ Mental Health Centre Copenhagen, Copenhagen, Mental Health Services, Capital Region of Denmark, Copenhagen, Denmark; ^2^ Mental Health Centre Sct. Hans, Roskilde, Mental Health Services, Capital Region of Denmark, Roskilde, Denmark; ^3^ Copenhagen Research Center for Mental Health – CORE, Mental Health Center Copenhagen, Copenhagen University Hospital, Copenhagen, Denmark; ^4^ Outpatient Clinics, Novavi Foundation, Frederiksberg, Denmark; ^5^ Psychiatric Center Amager, Mental Health Services, Capitol Region Hospitals, Amager, Denmark; ^6^ Department of Clinical Medicine, Faculty of Health and Medical Sciences, University of Copenhagen, Copenhagen, Denmark

**Keywords:** technology, addiction, psychotherapy, virtual reality, cognitive behavioral therapy, feasibility, alcohol, innovation

## Abstract

**Introduction:**

Cognitive behavioral therapy (CBT) is an evidence-based treatment for alcohol use disorder (AUD). Exposure to high-risk situations in virtual reality (VR) has been suggested to have a potential therapeutical benefit, but no previous study has combined VR and CBT for AUD. We aimed to investigate the feasibility of using VR-simulated high-risk environments in CBT-based treatment of AUD.

**Methods:**

We randomized ten treatment-seeking AUD-diagnosed individuals to three sessions of conventional CBT or VR-assisted CBT performed at two outpatient clinics in Denmark. In each session, patients randomized to VR-CBT were exposed to VR-simulations from a restaurant to induce authentic thoughts, emotions, physiological reactions, and craving for CBT purposes. The primary outcome measure was feasibility: Drop-out rate, psychological reactions, and simulator sickness. Secondary outcomes were assessment of preliminary short-term changes in alcohol consumption and craving from baseline to one-week and one-month follow-up. In addition, the study was conducted for training in operationalization of VR equipment, treatment manuals, and research questionnaires.

**Results:**

The majority of patients completed all study visits (90%). VR induced authentic high-risk related thoughts, emotions, and physiological reactions that were considered relevant for CBT by patients and therapists. Four of five patients randomized to VR-CBT experienced cravings during VR simulations, and most of these patients (3/5) experienced mild simulator sickness during VR exposure. The preliminary data showed that patients receiving VR-CBT had more reduction in alcohol consumption than patients receiving conventional CBT at one week- (median 94% vs. 72%) and one-month follow-up (median 98% vs. 55%). Similar results were found regarding changes in cravings.

**Conclusion:**

We demonstrated VR-CBT to be a feasible intervention for patients with AUD which supports continued investigations in a larger randomized clinical trial evaluating the efficacy of VR-CBT.

**Clinical trial registration:**

https://www.clinicaltrials.gov/study/NCT04990765?cond=addiction%20CRAVR&rank=2, identifier NCT05042180.

## Introduction

Alcohol Use Disorder (AUD) is a brain disorder that contributes to 3.3 million deaths each year globally (1, 2). According to the World Health Organization, approximately 5.3% of the global adult population is living with AUD (1). The disorder is linked to more than 200 diseases, injuries and other health conditions including mental and behavioral disorders, cancers, infections, and neurological diseases (3, 4). Beyond the health consequences, harmful use of alcohol negatively impacts the affected individual, relatives, and society (1, 4). Treatment options include pharmacological-, social- and psychotherapeutic interventions, but even when receiving full treatment, more than 60 percent of patients relapse within the first year following treatment (5, 6). Relapse has been linked to different biological- and psychological factors, including psychiatric co-morbidity, the severity of AUD, motivation, and craving (5). These factors are addressed in cognitive behavioral therapy (CBT), which is a well-established evidence-based treatment for patients with AUD (7).

CBT is a time-limited, multi-session intervention that targets cognitive, affective, and environmental risks for substance use (8, 9). The intervention is based on the theory that substance use disorders are characterized by maladaptive behavioral patterns and thinking derived from dysfunctional beliefs (9). For patients with AUD, dysfunctional beliefs are activated under high-risk situations and include anticipatory-, relief-oriented- and permissive beliefs (9). According to the cognitive model of substance use, the activation of dysfunctional beliefs is associated with corresponding thought patterns, ultimately resulting in a chain reaction towards relapse (9). CBT involves different techniques to modify dysfunctional psychological patterns and behavioral habits (8, 9).

Conventionally, CBT regimens involve exposure therapy (ET), where patients are repeatedly exposed to triggers to diminish an unwanted reaction, e.g., anxiety or craving (8). However, in the case of AUD, exposure has traditionally not been applied during CBT. The method is based on classical conditioning, where a neutral stimuli/cue, e.g., beer bottle, friends, or restaurant, eventually becomes a conditioned stimulus (CS) that can trigger craving and, thus, increase the risk of relapse (10). During ET patients are repeatedly exposed to cues to elicit cravings while refraining from consumption behavior, to weaken the association between the cue, craving, and consumption and hypothetically reduce the risk of craving-induced relapse (10). While the evidence for CBT is substantial, a meta-analysis only found minor effects of using ET for AUD (7, 11). However, when ET is combined with specific coping skill training, the combined treatment is more effective than conventional ET (11).

In addition to the limited evidence, it can be challenging to implement conventional ET in the treatment of AUD. First, it may be logistically difficult to bring alcohol into the treatment setting or accompany patients to their high-risk location. Secondly, it can be difficult to control the situation, and thirdly, conducting treatment in public spaces entails confidentiality issues (12). However, technological improvements have renewed interest in ET. Particularly virtual reality (VR) assisted ET has received increasing interest in mental health (13). Literature shows a variety of possible applications and promising results in reducing symptoms of anxiety, PTSD, psychosis, and eating- and addictive disorders, but the research is at an early stage (14–19).

In the field of AUD, VR has primarily been tested for exposure purposes, simulating conventional ET (18). VR-assisted ET can expose patients to cues in confidential, controllable, and safe clinical settings, and studies have found VR exposure to be more accepted by patients, less time-consuming, and easily repeated compared to conventional ET (12, 20–22). Available trials suggest that VR-assisted ET can reduce craving short term (18, 23–25). However, no study has investigated the effect of VR-assisted ET as an integrated part of CBT. The research field lacks methodological rigor, with small sample sizes and few randomized studies. All published studies have applied 3D-animated VR environments and often include projectors or computers for exposure instead of head-mounted devices, which may diminish immersion (18, 23).

Aiming to optimize treatment outcomes in patients diagnosed with AUD, we are preparing a larger randomized controlled trial to investigate the long-term efficacy of VR-assisted CBT. To this end, we have initially conducted the present feasibility study to investigate whether VR-assisted exposure of high-risk environments is realistic and therapeutically relevant as an integrated component of CBT-based treatment of patients with AUD.

## Materials and methods

This article is written in accordance with the Consolidated Standards of Reporting Trials (CONSORT) 2010: Recommendations for reporting randomized trials with the extension to randomized pilot and feasibility trials (26, 27). The protocol is registered at clinicaltrial.gov (https://clinicaltrials.gov/ct2/show/NCT04990765).

### Study design

The present study is an assessor-masked, randomized, controlled, parallel, clinical feasibility trial. The study was conducted from May 11^th^, 2021, to September 13^th^, 2021, and was designed to investigate the feasibility of VR-CBT vs. conventional CBT in 10 patients diagnosed with AUD.

### Participants and screening

**Figure 1:**
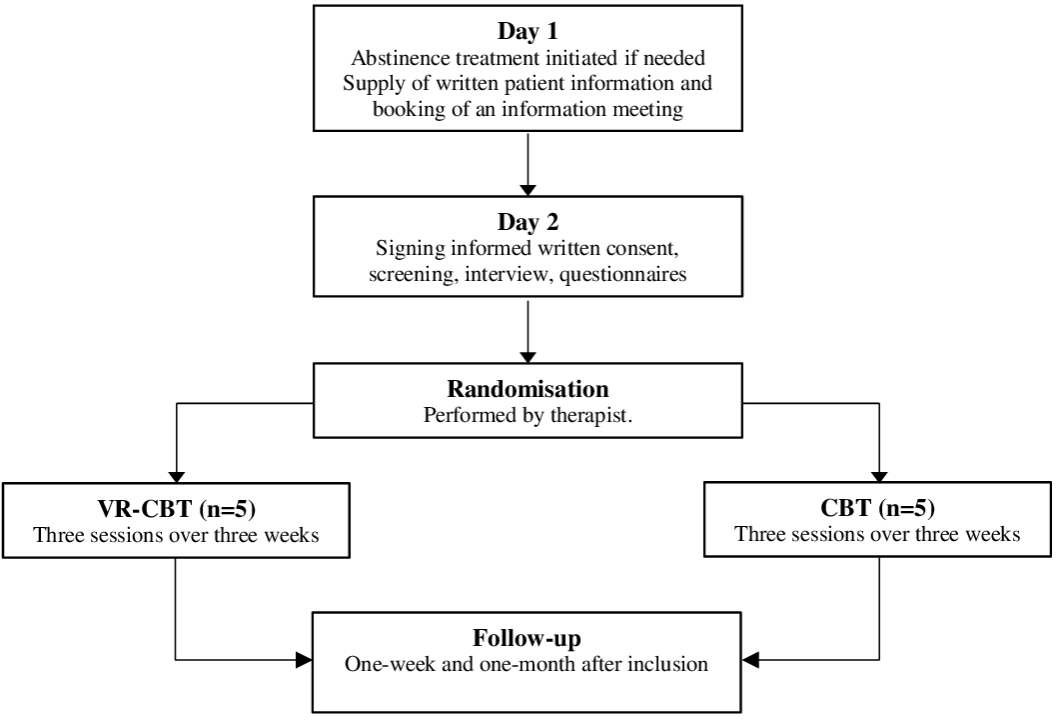
Study flow chart.

Patients were recruited from two outpatient clinics in the Capital Region of Denmark and Region Zealand, Denmark (www.novavi.dk). Patients could be referred from hospitals, general practitioners, communes, or self-referrals. Treatment was free of charge for everyone with a Danish Social Security Number. Each clinic had a medical doctor, therapeutically trained nurses, and social workers that initiated abstinence treatment if indicated, i.e., benzodiazepines and vitamins, and informed all patients about the clinical trial (**Figure 1**, Day 1). If the patient showed interest in participation, a researcher was contacted to schedule an information meeting. During the information meeting, the patient was informed verbally and in writing on rights and responsibilities while participating in the study (**Figure 1**, Day 2). A screening examination was performed after the patient had agreed to participate and had signed the informed consent form. At the time of screening, the patient was interviewed to ensure that all inclusion-, and none of the exclusion criteria were met. The patient was also asked about psychosocial factors in addition to treatment goals, motivation, alcohol history, previous treatment, family disposition, education, and civil status.

### Inclusion criteria

In order to be eligible for inclusion, patients had to give oral and written consent; be within the age range of 18-70 years old (both included); fulfill the diagnostic criteria for AUD (according to the criteria of ICD-10); and have had at least five days with heavy alcohol drinking in the 30 days prior to inclusion [defined as alcohol consumption over 60 g of alcohol per day (men) or 48 g of alcohol per day (women)].

### Exclusion criteria

To enable a thorough understanding of feasibility while limiting cofounding relationships, the following exclusion criteria were used: Patients were excluded from participation if they were diagnosed with any severe neuropsychiatric disease, e.g., schizophrenia, paranoid psychosis, bipolar disorder, or substantial cognitive impairment; had any other active substance use [defined as a Drug Use Disorders Identification Test (DUDIT)-score >6 (men) >2 (women) and fulfilling the criteria for a dependence of the substance according to the criteria of ICD-10 (except nicotine)]; had received any pharmacological treatment targeting AUD, i.e., acamprosate, disulfiram, naltrexone, or nalmefene, within the last 30 days and up to one-month follow-up; or were unable to speak or understand Danish.

### Intervention

CBT sessions were scheduled once weekly for three weeks for each patient in both study arms and performed by therapeutically educated and registered nurses. Each session lasted 45-60 minutes and was structured similarly, as presented in **Appendix 1**. Treatment sessions were based on a manualized treatment protocol. Session 1 focused on psychoeducation about addiction, introduction to the cognitive method, and identification of high-risk situations. Session 2 focused on motivation and continuation on the introduction to the cognitive method. Session 3 aimed at identifying problems and goals (specific, measurable, attainable, relevant, and time-based), and evaluating pros and cons on reduction/abstinence vs. status quo. Session homework was cognitive analysis of a specific situation (session 1), and registration of craving for seven days (session 2), whereas no homework was scheduled after session 3. To ensure a standardized treatment regimen for both study arms, all nurses received 49 hours of education and training before initiation of the study. During the study, nurses also received supervision based on experiences during treatment sessions. To ensure a high degree of fidelity, nurses used the treatment manual outlined in **Appendix 1**. During sessions, the therapist and the patient collaboratively used the treatment manual to structure sessions and gather information on reactions during VR exposure. The therapists collected data in the manuals which ensured adherence to the protocol.

### Conventional CBT vs. VR-CBT

**Figure 2:**
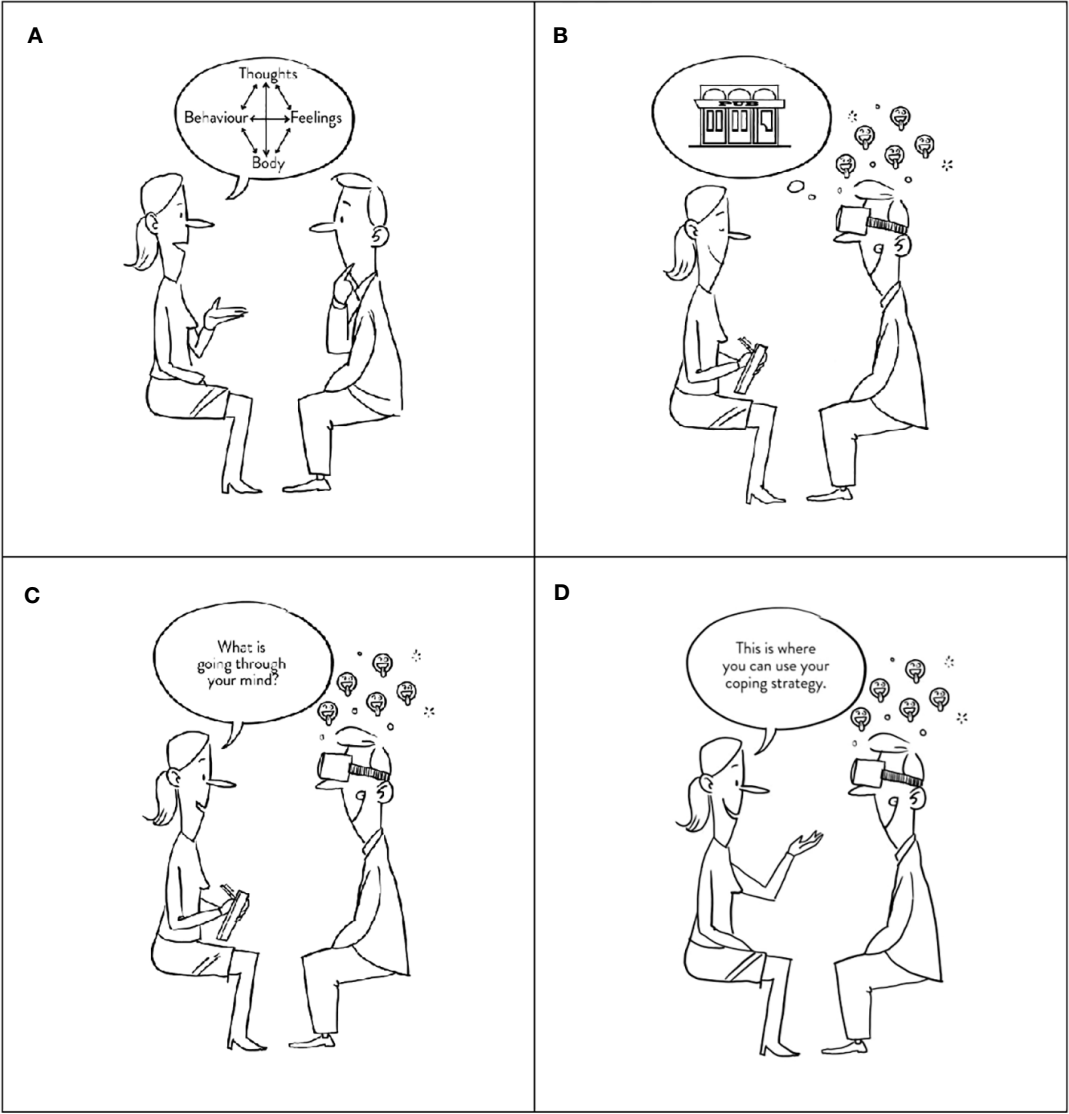
Interventions. The figure shows the main difference between the two study arms. **(A)** illustrated a fundamental element of CBT, the cognitive analysis, whereas **(B)** illustrates a patient exposed to a high-risk situation which induces craving (emojis). During exposure, the therapist then asks the patient what goes through his mind for cognitive analysis, either performed during VR exposure **(C)** or after exposure, as illustrated in **(A)**. Finally, the nurse instructs the patient to practice a new coping strategy **(D)**.

**Figure 3:**
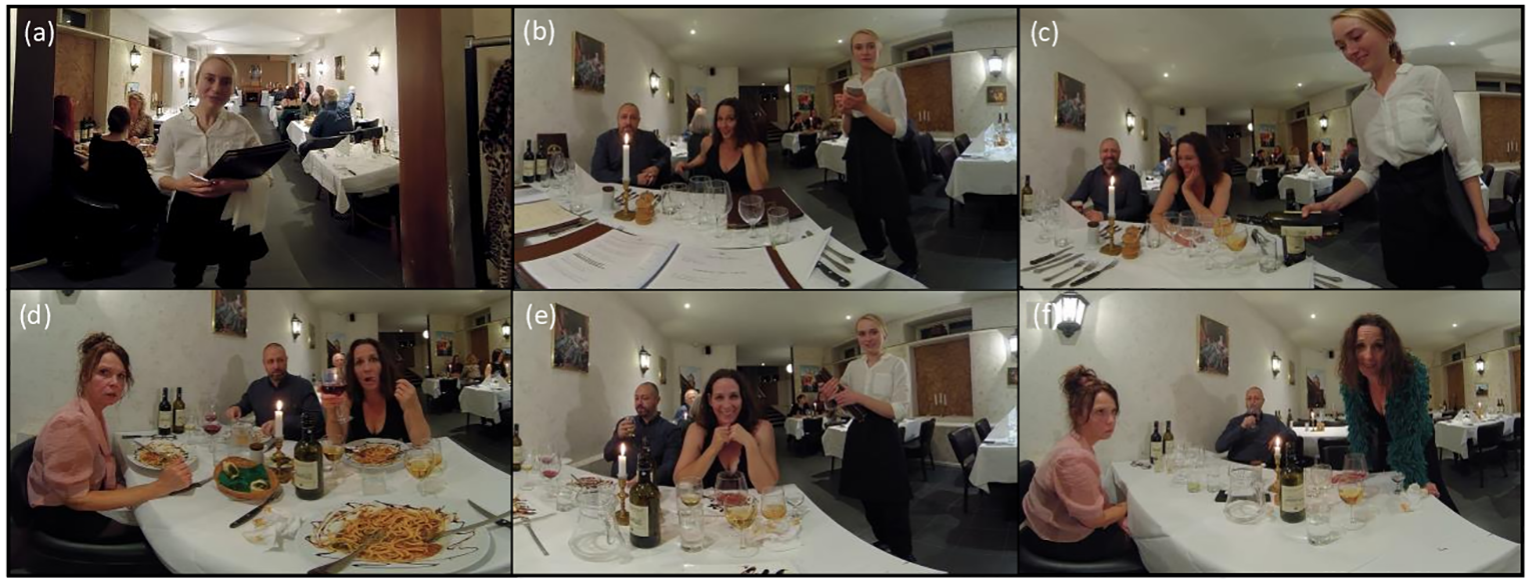
VR high-risk restaurant scenes. The figure shows the six different restaurant scenes used for VR exposure. **(A)** Arrival at a restaurant (1:39 mins), **(B)** Ordering food and drinks (3:53 mins), **(C)** Drinks are being served (3:23 mins), **(D)** Drinking problem is revealed (3:55 mins), **(E)** Friend offers shots (3:52 mins), **(F)** Friends want to go out (2:13 mins). Scenes **(A, C, E)** were used for cognitive analysis, while scenes **(B, D, F)** were used for coping skill training.

Patients in both study arms performed all tasks mentioned above and in **Appendix 1**. However, cognitive analysis and coping strategies were performed differently between study arms, as shown in **Figure 2**. For cognitive analysis, patients randomized to conventional CBT choose a specific anecdotal high-risk scenario to identify thoughts and emotional-, physiological-, and behavioral responses (**Figure 3**: videos **A, C, E**). In contrast, patients randomized to VR-CBT were exposed to a pre-determined VR high-risk restaurant scene. The scene constituted the basis for cognitive analysis during or after the VR exposure.

After the cognitive analysis, patients in the conventional CBT study arm were informed about coping strategies to use when experiencing cravings. Contrary, patients randomized to VR-CBT were informed about the coping strategy and then instructed to practice it during the next pre-determined VR high-risk restaurant scene (**Figure 3**: videos **B, D, F**).

The following three evidence-based coping strategies were used in both treatment arms.

• Session 1: Think about the negative consequences of alcohol consumption and positive consequences of reduction or abstinence (depending on goals).
• Session 2: Distraction from craving by thinking- or focusing on something else.
• Session 3: Breathing exercise (focus on breathing).

Patients randomized to VR-CBT registered VR-induced simulator sickness at the end of each treatment session using the Simulator Sickness Questionnaire (SSQ).

### Withdrawal criteria

Patients were free to withdraw from the trial at any time without providing a reason, and they were informed that withdrawal would have no impact on further treatment at the Novavi outpatient clinics. The reason for withdrawal was registered if the patient wished to inform the study personnel. As this was a feasibility trial with only 3 therapeutic sessions, failure to comply with the clinical trial protocol, i.e., if the patient missed more than one treatment session in total, resulted in being withdrawn from the study.

### VR high-risk environments

Patients randomized to VR-CBT were exposed to 360-degree environments from a restaurant. All environments were filmed with a GoPro Camera® (www.gopro.com) and produced by movie producer and instructor, Anne Heeno, using professional actors and extras (www.timestoryvr.com). As presented in **Figure 3**, the high-risk environments consist of six different environments from a restaurant (graded 1-6) that gradually increases in alcohol-related cues, emotional triggers, and interaction with actors. Environments graded 1 to 4 primarily focus on alcohol cues and emotional triggers, while environments graded 5 and 6 also have an interactive element, where actors (avatars) either ask the patient if he/she wants a beverage (patients are instructed to say no) or confront the patient with alcohol-related topics. All environments were designed to activate high-risk related thoughts, emotions, and physiological reactions, including craving. The title of each environment is related to the content and includes: “Arriving at a restaurant”, “Ordering food and drinks”, “Drinks are being served”, “Drinking problem is revealed”, “Friend offers shots”, and “Friends want to go out” (**Figure 3**).

The present study precedes a clinical RCT investigating the efficacy of VR-CBT in patients with AUD (28). Based on Ghiţă et al. questioning patients about locations that trigger cravings, we produced VR environments from five different locations: pub, bar/party, restaurant, at-home, and supermarket (29). Restaurant environments were the first available scenes and thus used in the present feasibility study.

### Quantitative assessment

At inclusion, one week, and one month after the third- and final treatment session, masked assessor evaluations were performed on alcohol consumption using Timeline Follow back (TLFB) (29) and Global Assessment of Functioning (GAF) (30), while unmasked patient reported assessments were conducted on cue-induced craving before, during (maximum), and after each VR-Exposure evaluated on a Visual Analogue Scale (0 = no craving and 10 = most intense imaginable craving) (31), Penn Alcohol Craving Scale (PACS) (32), Alcohol Use Disorders Identification Test (AUDIT) (33), Drug Use Disorders Identification Test (DUDIT) (34), Beck Depression Inventory-II (BDI-II) (35), Beck Anxiety Inventory (BAI) (36). To determine simulator sickness from VR, the self-reported Simulator Sickness Questionnaire (SSQ) was used unmasked (37).

### Sample size and randomization

A sample of ten patients was chosen for each nurse to practice the CBT manuals for both study arms. When a patient was recruited and included by the investigators, the secretary at each of the two outpatient clinics would schedule the first session with the first available therapist. Similar to other multicenter feasibility studies, patients were randomized in blocks of two and allocated 1:1 to either CBT or VR-CBT (38). Randomization was stratified by therapist to minimize any confounding effects of specific therapists and for each therapist to practice manuals for both study arms (38). Each therapist conducted the randomization by opening one of two sealed envelopes allocating the patient to either CBT or VR-CBT. When the therapists later scheduled their second patient, the patient was allocation into the other study arm. Randomization was registered in the randomization module in Research Electronic Data Capture (REDCap). While investigators performing assessments and data analysis remained masked until the database unlocked, patients and therapists were unmasked.

### Statistics

Due to the nature of the study, i.e., feasibility study and the small sample size (n=10), no statistical analysis was performed. Data are reported descriptively using medians and range from minimum to maximum. VR-induced reactions (thoughts, emotions, physiological reactions), craving levels before, during, and after VR exposure, and VR-induced simulator sickness (SSQ), were collected from patients randomized to VR-CBT. Data from the remaining questionnaires were collected from participants in both study arms.

### Outcome measures: feasibility and preliminary efficacy

The primary outcome measure was feasibility measured through drop-out rates, VR-induced reactions (thoughts, emotions, psychological reactions incl. cravings), and simulator sickness. The secondary outcome measures on preliminary efficacy included percentage change from inclusion to one- and four weeks FU in total alcohol consumption, HDD, PACS, AUDIT, DUDIT, BAI, BDI, and GAF.

### Ethical considerations

The study is approved by The Regional Ethics Committee (journal number H-20082136) and The Danish Data Protection Agency (protocol number RHP-2021–217). On ClinicalTrials.gov, ethical considerations can be identified by the ID NCT05042180. The protocol has version control and dates as identifiers.

## Results

### Demographics

A total of nine patients completed all treatment- and follow-up visits, while one patient was excluded before the first treatment session due to the initiation of disulfiram (exclusion criterion). Demographics are presented in **Table 1**. The median age was 46 years, genders were almost equally distributed (56% male), and most patients were married or with a partner (67%).

**Table 1:** Demographics.

	ID	Gender	Age	Fam. disp.	Alcohol consumption debut (age)	Marital status	ICD-10	Previous TX	Excessive consumption	TX Goal
CBT	2	Male	46	Yes	14	Married	6	No	22 years	Reduction
	4	Male	46	Yes	13	Married	6	Yes	2 years	Reduction
	5	Male	22	Yes	16	Single	6	No	3 years	Reduction
	8	Female	59	Yes	17	Single	6	Yes	10 years	Reduction
VR-CBT	1	Female	54	No	16	Partner	6	Yes	7 years	Abstinence
	3	Male	38	Yes	11	Married	4	No	22 years	Reduction
	6	Male	42	Yes	14	Married	4	No	10 years	Reduction
	7	Female	61	Yes	15	Single	6	No	6 years	Reduction
	9	Female	67	No	16	Married	6	No	10 years	Abstinence

### Alcohol history and comorbidities

Patients debuted with alcohol consumption at a median age of 15 years (range 11-17). They reported consuming alcohol to regulate emotions (n=9), including sadness, loneliness, and anxiety, be more social (n=5), remove withdrawal symptoms (n=2), remove pain (n=1), and for pleasure (n=1). Of the nine patients, the majority were dispositioned to AUD (7/9) and had not received treatment for AUD previously (6/9). All patients were diagnosed with AUD at inclusion, and most reported all six symptoms of dependency (7/9), according to ICD-10. The remaining two patients reported four symptoms of dependency at inclusion. No psychiatric comorbidities were reported, while the following physical disorders were reported: abdominal hernia (ID2), herniated disc (ID7), back pain (ID8), osteoarthritis (ID8, ID9), and psoriasis (ID9).

### Motivation

At inclusion, the treatment goal was reduction/moderation for the majority (7/9) and abstinence for the two remaining patients. In terms of motivation for seeking treatment, most patients mentioned improvement in physical- and mental health (n=8), followed by family (n=5), getting more energy (n=2), not lying (n=1), and regaining a driver’s license (n=1). All patients graded the importance of achieving their treatment goal to ten on a ten-point scale. On the contrary, six patients graded their belief in successfully achieving their goal to ten points, two to eight points, and one to nine points on a ten-point scale.

### Patient-reported high-risk scenarios

Patients reported high-risk scenarios regarding locations, situations, and emotions at the first treatment session. The most frequently reported locations were at parties (n=6), restaurants (n=2), at home (n=2), and supermarkets (n=1). High-risk situations were after work (n=4), meeting friends (n=2), days off (n=1), holidays (n=1), when music was playing (n=1), pain (n=1), and when meeting new people at social events (n=1). Patients reported sadness (n=5), anger (n=3), anxiousness (n=3), happiness (n=2), loneliness (n=2), and boredom (n=2) as high-risk emotions.

### VR-induced craving

**Figure 4:**
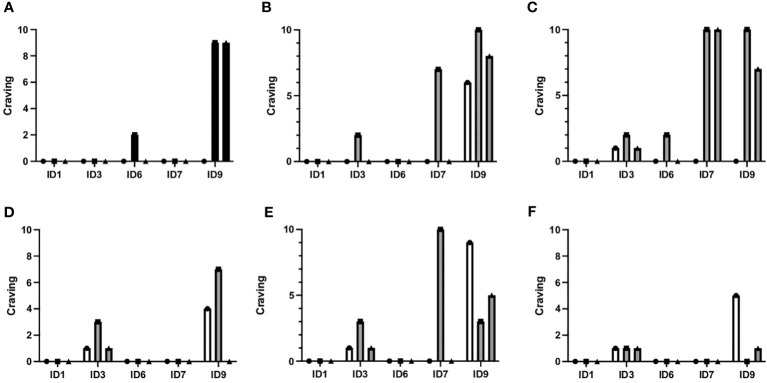
Craving before, during and after each VR exposure. The figure shows craving levels before, during, and after all six different VR restaurant scenes for each patient randomized to VR-CBT (ID1, ID3, ID6, ID7, and ID9). **(A)** Arrival at restaurant, **(B)** Ordering food and drinks, **(C)** Drinks are being served, **(D)** Drinking problem is revealed, **(E)** Friend offers shots, **(F)** Friends want to go out. The graphs correspond to the videos in **Figure 3**. Scenes **(A, C, E)** were used for cognitive analysis, while scenes **(B, D, F)** were used for coping skill training.

Almost all patients experienced cravings during VR exposure (4/5), as illustrated in **Figure 4**. Interestingly, one patient reported no craving during any exposures (ID1), while two patients experienced low craving levels (ID3, ID 6), one patient had moderate craving levels (ID7), and one patient had high craving levels (ID9). Overall craving scores during VR exposure were generally low, with a median of 2 (range 0-10).

For each VR-simulated restaurant environment, maximum craving scores were the following: Environment 1 (a): median 0 (range 0-9), Environment 2 (b): median 2 (range 0-10), Environment 3 (c): median 2 (range 0-10), Environment 4 (d): median 0 (range 0-7), Environment 5 (e): median 3 (range 0-10), and Environment 6 (f): median 0 (0–1).

### VR-CBT-induced reactions (cognitive analysis)

During VR exposure, patients reported thoughts, emotions, and physiological reactions, as presented in **Appendix 2**. Thoughts were primarily related to alcohol cues, the people at the restaurant table, and the peer pressure to take a shot. Emotions ranged from irritation to satisfaction, happiness, frustration, disgust, confusion, cravings, anger, sadness, helplessness, and surprise. Thus, emotions induced by VR exposure included emotions reported as triggers by patients, i.e., sadness, anger, and happiness. The physiological reactions reported during sessions were relaxation, tension, agitation, restlessness, headache, and stomach pain (**Appendix 2**).

### Simulator sickness

Most patients reported transient simulator sickness during VR exposure (3 of 5 patients): Mild general discomfort (n=3), mild tiredness (n=2), mild and moderate headache (n=2), mild dizziness with open eyes (n=1), mild eye discomfort (n=1), moderate difficulties focusing (n=1), unclear vision (n=1), mild fullness of the head (n=1), and mild to moderate stomach awareness (n=1).

### The total reduction in alcohol consumption

**Figure 5:**
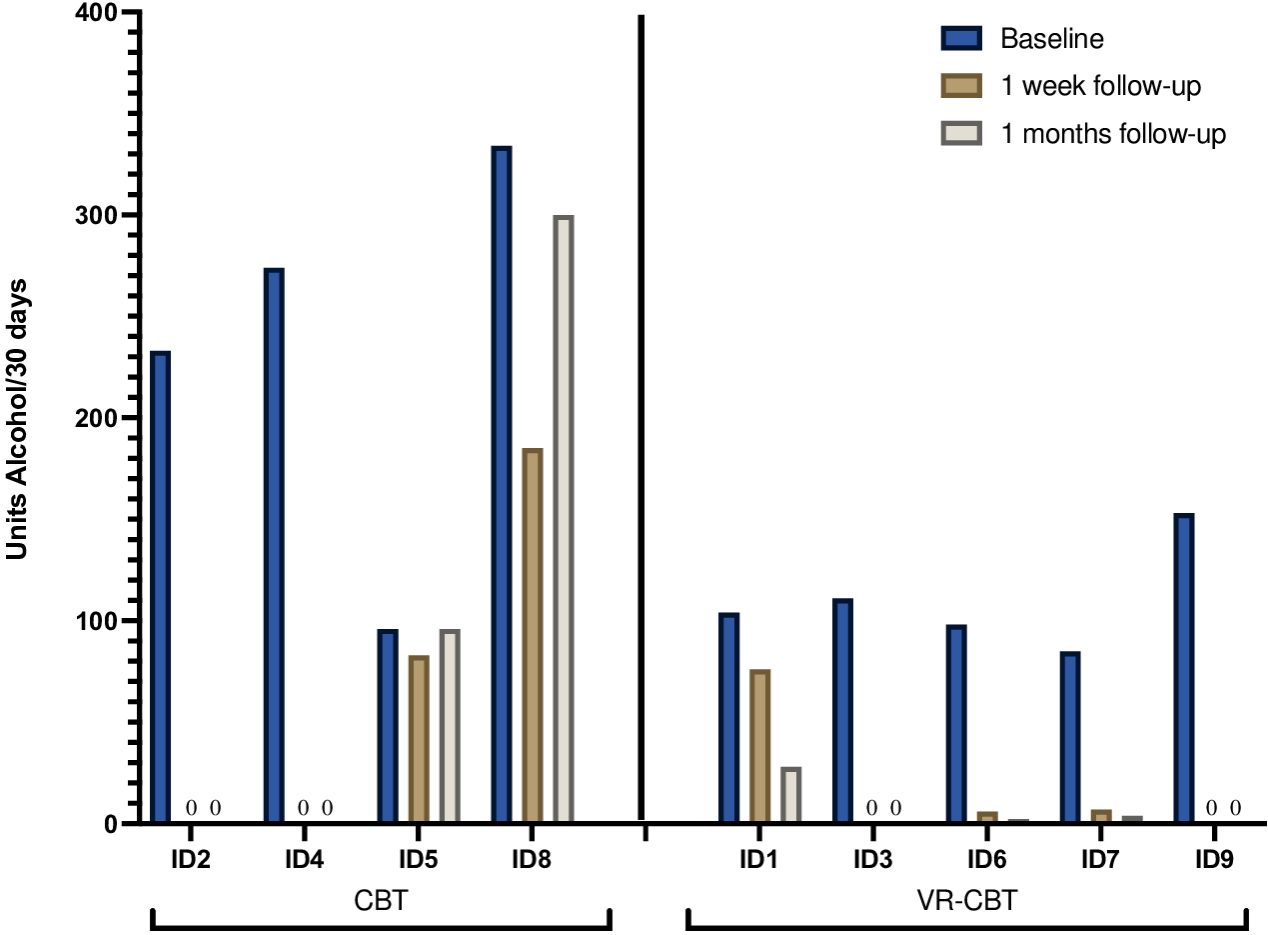
Alcohol consumption.

The course of alcohol consumption from baseline to one week- and one-month FU is illustrated for each patient in **Figure 5**. At baseline, patients reported consumption of median 111 alcohol units (range 85-334 units) during the last 30 days. Alcohol consumption at baseline was higher for patients randomized to CBT (median 253.5, range 96-334) compared to patients randomized to VR-CBT (median 104, range 85-153). For patients randomized to conventional CBT, abstinence was achieved by 2/4 patients (ID2, ID4), while one patient (ID8) achieved a 44% reduction at one-week FU, declining to a 10% reduction at one-month FU. The final patient (ID5) had a 14% reduction at one-week FU, which returned to baseline levels at one-month FU. In the VR-CBT group, abstinence was achieved by 2/5 patients (ID3, ID9), while 2/5 patients (ID6, ID7) achieved >90% reduction at both FU visits, and the final patient (ID1) had a 27% reduction at one-week FU increasing to 73% reduction at one-months FU. No patient experienced an increase in alcohol consumption.

When data from all patients were pooled, the median reduction in alcohol units was 94% at one week- (range 14-100%) and 98% at one month FU (range 0-100%) (**Table 2**). When reduction was compared between the two interventions, the median reduction was greater in patients randomized to VR-CBT at one-week FU (94% vs. 72%) and one-month FU (98% vs. 55%) (**Table 2**).

**Table 2:** Secondary outcomes.

	All patients (n=9)		CBT (n=4)		VR-CBT (n=5)	
	Score	Change (%)	Score	Change (%)	Score	Change (%)
Total alcohol consumption (units)						
Baseline	111 (85-334)		253.5 (96-334)		104 (85-153)	
1 week FU	6 (0-185)	94% (14-100)	41.5 (0-185)	72% (14-100)	6 (0-76)	94% (27-100)
1 month FU	2 (0-300)	98% (0-100)	48 (0-300)	55% (0-100)	2 (0-28)	98% (73-100)
Heavy drinking days						
Baseline	17 (5-30)		17.5 (5-30)		15 (9-28)	
1 week FU	0 (0-30)	100% (-80-100)	4.5 (0-30)	50% (-100-80)	0 (0-14)	100% (42-100)
1 month FU	0 (0-30)	100% (-220-100)	8 (0-30)	50% (-100-220)	0 (0-7)	100% (71-100)
PACS						
Baseline	25 (7-27)		25 (19-26)		15 (7-27)	
1 week FU	11 (4-26)	43% (-4-56)	15 (9-26)	41% (-53-4)	8 (4-22)	43% (19-56)
1 month FU	8 (0-30)	47% (-15-100)	17.5 (0-30)	30% (-100-15)	7 (6-22)	47% (0-74)
AUDIT						
Baseline	26 (16-34)		30.5 (22-34)		24 (16-27)	
1 week FU	19 (12-27)	25% (0-35)	22 (19-27)	26% (0-33)	17 (12-20)	25% (13-35)
1 month FU	19 (11-29)	25% (0-39)	25.5 (17-29)	13% (0-39)	19 (11-20)	26% (21-31)
DUDIT						
Baseline	0 (0-2)		0 (0-2)		0 (0-0)	
1 week FU	0 (0-0)	0% (0-100)	0 (0-0)	0% (0-100)	0 (0-0)	0% (0-0)
1 month FU	0 (0-0)	0% (0-100)	0 (0-0)	0% (0-100)	0 (0-0)	0% (0-0)
GAF						
Baseline	80 (60-90)		65 (60-90)		90 (80-90)	
1 week FU	90 (60-100)	0% (-25-50)	85 (70-90)	+25% (-11-50)	90 (60-100)	0% (-13-25)
1 month FU	90 (70-100)	+11% (-13-50)	90 (90-90)	+39% (0-50)	90 (70-100)	+11% (-13-13)
BDI-II						
Baseline	13 (3-28)		24.5 (13-28)		10 (3-28)	
1 week FU	9 (3-21)	27% (-70-100)	14 (7-20)	30% (27-70)	6 (3-21)	25% (-25-100)
1 month FU	5 (0-30)	25% (-100-200)	13-5 (5-24)	41% (8-78)	5 (0-30)	25% (-50-200)
BAI						
Baseline	17 (1-23)		19 (10-23)		4 (1-22)	
1 week FU	4 (1-24)	65% (-88-14)	6 (2-24)	63% (-88-14)	1 (1-12)	67% (0-75)
1 month FU	3 (0-14)	78% (0-100)	2.5 (0-14)	89% (33-100)	3 (0-13)	41% (0-100)

AUDIT, Alcohol Use Disorders Identification Test; BAI, Beck Anxiety Inventory; BDI-II, Beck Depression Inventory-II; DUDIT, Drug Use Disorders Identification Test; GAF, Global Assessment of Functioning; PACS, Penn Alcohol Craving Scale.

### Heavy drinking days

At baseline, the median number of HDD was 17 (range 5-30) for all patients, which was almost similar when looking separately at each study arm: CBT (median 17.5, range 5-30) vs. VR-CBT (median 15, range 9-28). When data from all patients were pooled, the median reduction in HDD was 100% at one week- (range +80% to -100%) and 100% at one month FU (range +220% to -100%) (**Table 2**). However, when comparing study arms, the median reduction in HDD was twice as high in patients randomized to VR-CBT at one-week FU (100% vs. 50%), and one-month FU (100% vs. 50%), compared to patients randomized to conventional CBT (**Table 2**).

### PACS

**Table 2** shows craving levels at baseline, one week-, and one month FU for all patients and both study arms. At baseline, the median craving level was 25 (range 7-27), and craving levels were higher in patients randomized to CBT (median 25, range 19-26) vs. VR-CBT (median 15, range 7-27). For patients receiving CBT, the median reduction in craving from baseline to one-week FU (41%, range +4% to 53%) was almost similar to patients randomized to VR-CBT (43%, range 19-56%). However, at one-month FU, the median reduction in craving levels was higher after VR-CBT (47%, range 0-74%) vs. conventional CBT (30%, range +15 to 100%).

### AUDIT, DUDIT, GAF, BDI and BAI

At baseline, patients randomized to conventional CBT had a greater AUDIT score (30.5 vs. 24), lower general functioning level (65 vs. 90), as well as greater BDI-II- (24.5 vs. 10) and BAI score (19 vs. 4), compared to patients randomized to VR-CBT. When data from all included patients were pooled, considerable reductions were found in AUDIT, BDI, and BAI, whereas no or only minor changes were found in DUDIT or GAF (**Table 2**). Patients randomized to conventional CBT achieved a greater increase in general functioning level at one-week FU (25% vs. 0%) and one-month FU (39% vs. 11%), compared to VR-CBT. Similar tendencies were found in the reduction of BDI-II- and BAI scores, where patients randomized to conventional CBT achieved a greater reduction.

## Discussion

To our knowledge, the present study is the first to investigate the feasibility of VR-CBT in treating patients with AUD. Our findings indicate that VR-CBT is a feasible intervention in terms of tolerability and acceptability by the patients, and capability of VR environments to induce authentic psychological reactions for therapeutic purposes.

Adherence to treatment is essential for feasibility, and a meta-analysis including 21 studies of AUD-diagnosed patients receiving psychosocial treatment reports an average drop-out rate of 26.1% (39, 40). In the present study, the five patients randomized to VR-CBT and four of five patients randomized to conventional CBT completed all study visits, with an overall drop-out rate of 10%. Due to the study aim, i.e., feasibility, the investigation only included three treatment sessions and short-term follow-up (1 month), which may explain the low drop-out rate in the present study. Nevertheless, the low drop-out rate is notable in the context of more than half of patients randomized to VR-CBT experiencing simulator sickness during VR exposure. Since patients experienced both cravings and emotional responses during exposure to VR environments, this might have intensified simulator sickness. However, this finding is consistent with previous studies reporting that simulator sickness during VR only have minor impact on adherence to treatment in populations with mental illness (41). Simulator sickness is generally underreported in AUD-diagnosed populations exposed to VR environments making these findings particularly noteworthy (18).

Craving is considered an important treatment target in patients with AUD and several studies have demonstrated that VR environments can induce and reduce cravings in this population (23). Interestingly, all previous studies have exposed patients to 3D animated environments mostly from computer screens or projectors, while only one study has used head mounted devices (18). To address these limitations, we exposed patients to complex VR environments through head mounted devices using real-life locations and interactions with professional actors/avatars. Our results substantiate the previous findings, however, craving levels during VR exposure were generally low (median 2, range 0-10): one patient experienced high craving levels, three patients experienced moderate to low craving levels, and only one patient reported no craving during any exposure. We only exposed patients to VR environments from a restaurant which may explain the limited craving levels during VR exposure. This finding implies the importance of having access to a broad range of high-risk environments to make the intervention relevant and feasible for different patients.

To our knowledge, this is the first study to show that VR environments can induce a wide range of thoughts, emotions, and physiological reactions. Among others, these included anxiety, anger, happiness, sadness, disgust, tension, agitation, cravings, and alcohol-related thoughts, i.e., *“I wish I was her right now, she can drink, and I wish I could as well”*, and *“It could be nice to drink something with the others”*. Although it has previously been reported that emotional responses may enhance cravings, one patient experienced a decrease in cravings while feeling irritation and helpless from being pressured to drink in the VR environments (42). Her thoughts included: “*I am alone. I am not a part of their connection. They don’t understand me. They are mean. I can’t have these friends anymore. They are not my friends. They will not decide for me”.* Thus, emotional responses may also reduce cravings. Interestingly, the same patient expressed experiencing strong alcohol cravings (VAS 10) and urges to take the wine glass out of an actors/avatars hand and drink the wine. Later the patient reported that these experiences made her realize the severity of her dependency which increased her motivation to remain abstinent. Based on these data we hypothesize that exposure to complex VR environments may accelerate insight of psychological and behavioral patterns with a corresponding therapeutic benefit.

Finally, previous studies investigating the efficacy of VR exposure in AUD-diagnosed patients have primarily evaluated changes in cue-reactivity (25). Therefore, we designed and conducted the present feasibility study as a predecessor to a larger randomized clinical trial investigating the long-term effect of VR exposure on alcohol consumption as the primary outcome (28). Thus, the data presented in the present study are preliminary and highlight the feasibility of the novel intervention, the equipment (head-mounted devices), treatment manuals, questionnaires, safety, attrition, and the ability of VR environments to induce therapeutically relevant reactions. The preliminary data from assessor-masked evaluations performed at one-week and one-month follow-up visits showed a remarkable reduction in alcohol consumption, heavy drinking days, cravings, and symptoms of depression and anxiety in both study arms. When results from the two study arms (CBT vs. VR-CBT) were compared, patients randomized to VR-CBT had a greater median reduction in total alcohol consumption, HDD, and cravings. On the contrary, patients randomized to conventional CBT experienced greater improvements in general functioning and symptoms of depression and anxiety. However, these data are preliminary and based on a small sample limited by an uneven distribution of patients into the two treatment arms. Patients randomized to conventional CBT generally consumed more alcohol, experienced more cravings, and had more symptoms of depression and anxiety, and lower functioning levels at baseline compared to patients randomized to VR-CBT. It is further important to emphasize that patients only received three treatment sessions. Accordingly, no statistical analysis was conducted, and the treatment regimen should not be considered sufficient or comparable to a standard treatment program.

The present feasibility study has several strengths, including adherence to the CONSORT 2010 statement: extension to randomized pilot and feasibility trials, pre-registration of the study protocol, inclusion of a head mounted device, the development and application of complex VR environments with professional actors/avatars, education and supervision of CBT-trained nurses, and assessment of changes in craving and alcohol consumption up to one month follow-up. Also, the limitations pertaining to the feasibility study should be mentioned: only six different VR environments from a restaurant was available; the population was limited to include treatment-seeking individuals with AUD and no severe neuropsychiatric disease; randomization was unsuccessful with heterogeneity regarding alcohol consumption, cravings, and demographics, i.e. comparison of two different populations; only 10 patients were included and received three CBT session with a short follow-up period; no objective assessments of craving or consumption were conducted; presence during VR exposure was not evaluated. To address the limitation, we are currently conducting a larger RCT that includes 30 different VR environments from a pub, party, restaurant, supermarket, and at-home, where patients are scheduled for a total of 14 treatment sessions. The treatment efficacy will be evaluated quantitatively (craving and alcohol consumption) and qualitatively (patients and therapists) up to 12 months after inclusion. To ensure homogeneity between the two study arms we have included electronic randomization using stratification for alcohol consumption, age, and gender (28). In conclusion, we have demonstrated VR-CBT to be a feasible intervention for patients with AUD, and the preliminary findings support further investigation of the efficacy of VR-CBT in patients with AUD using RCT and other rigorous methodology.

The present work combines VR and CBT as an initial step in exploring the possibilities of immersive technologies within an evidence-based treatment framework of AUD. On the horizon lies incorporation of augmented reality (AR) that combines elements from the physical world with computer-generated information in the same visual field. Contrary to VR, this creates an opportunity for patients to visually interact with the therapist while also being exposed to high-risk situations and alcohol-related cues. To our knowledge, no published study has investigated AR as a tool to improve the treatment of patients with AUD. We propose that future studies will assess VR- and AR-assisted interventions to ultimately improve the treatment of patients with AUD and other substance use disorders; a population that is currently underserved.

## Data availability statement

The raw data supporting the conclusions of this article will be made available by the authors, without undue reservation.

## Ethics statement

The study is approved by The Regional Ethics Committee (journal number H-20082136). The studies were conducted in accordance with the local legislation and institutional requirements. The participants provided their written informed consent to participate in this study. Written informed consent was obtained from the individual(s) for the publication of any potentially identifiable images or data included in this article.

## Author contributions

DT: Conceptualization, Data curation, Formal analysis, Funding acquisition, Investigation, Methodology, Project administration, Visualization, Writing – original draft, Writing – review & editing. SH: Conceptualization, Investigation, Methodology, Writing – review & editing. AV: Writing – review & editing. IO: Methodology, Writing – review & editing. MN: Supervision, Validation, Writing – review & editing. SD: Conceptualization, Methodology, Supervision, Validation, Writing – review & editing. AF: Conceptualization, Methodology, Supervision, Writing – review & editing.
