# Supplementary material for: Virtual reality-assisted cognitive behavioral therapy for patients with alcohol use disorder: a randomized feasibility study
Source: Front Psychiatry. 2024 Feb 14;15:1337898. doi: 10.3389/fpsyt.2024.1337898 (PMC10899342; doi:10.3389/fpsyt.2024.1337898)
Supplement: Supplementary file 1 [file DataSheet_1.docx]

**Appendix 1** Content and structure of manualized CBT-sessions

1. Mood check
   1. “When you think about the last week…
      1. How have you been personally?
      2. How have you been in your close relationships?
      3. How have you functioned socially?
      4. Looking at your answers, how will you evaluate your life in general?
2. Set the agenda together
3. Homework from last session (Table 1)
4. Today’s topic
   1. Conventional CBT:
      1. Cognitive analysis
      2. Coping strategy
      3. Topic of the day (Table 1)
   2. VR-CBT:
      1. VR exposure:
         1. Video 1: Cognitive analysis
         2. Video 2: Coping strategy training
      2. Topic of the day (Table 1)
5. New homework (Table 1)
6. Summary, feedback, and evaluation of VR-induced simulator sickness
   1. Content from the session is summarized
   2. “When you think about our conversation…
      1. To what degree did you feel heard and understood?
      2. To what degree did we work with topics you wished to work on?
      3. To what degree did you think that the way I (the therapist) work makes sense and fits you?
      4. Looking at your answers collectively, how will you evaluate today’s session?
   3. VR-CBT: Simulator sickness evaluated using Simulator Sickness Questionnaire (SSQ)
