# Supplementary material for: Virtual reality-assisted cognitive behavioral therapy for patients with alcohol use disorder: a randomized feasibility study
Source: Front Psychiatry. 2024 Feb 14;15:1337898. doi: 10.3389/fpsyt.2024.1337898 (PMC10899342; doi:10.3389/fpsyt.2024.1337898)
Supplement: Supplementary file 2 [file DataSheet_2.docx]

**Appendix 2:** VR-related cognitive analysis and corresponding craving levels

|  | **ID** | **SCENE 1: ARRIVING AT THE RESTAURANT** | **SCENE 3: DRINKS ARE BEING SERVED** | **SCENE 5: FRIENDS OFFER SHOT** |
| --- | --- | --- | --- | --- |
| **THOUGHTS** | **ID1** | *The cold wine looks nice. I like wine with ice cubes. I choose not to drink.* | *The wine looks delicious. The glass I received a week ago did not taste well. I'm getting heavy-headed. I'll leave after this.* | *The woman is extremely annoying. She insisted  that I should drink. I want to get away. It’s not fair to pressure me. She is thoughtless.* |
|  | **ID3** | *I wanted to ask if they had anything alcohol-free.* | *It could be nice to drink something with the others.* | *Please understand no for an answer* |
|  | **ID6** | *The music is annoying.* | *Fake company. Who are these people? I would rather have a beer than wine.* | *Stop pressuring me* |
|  | **ID7** | *-* | *-* |  |
|  | **ID9** | *I'm gonna take the wine out of her hand and drink it.*  *I need it now.* | *She could be me. She has been drinking from home. I wish I were her right now. She can drink, and I wish I could as well.* | *I am alone. I am not a part of their connection. They don’t understand me. They are mean. I can’t have these friends anymore. They are not my friends. They will not decide for me.* |
| **EMOTIONS** | **ID1** | *Satisfaction* | *Disgusted* | *Irritation and sadness* |
|  | **ID3** | *-* | *Craving* | *Irritation* |
|  | **ID6** | *Happiness, irritation and surprise* | *Happy and confusion* | *Anger* |
|  | **ID7** | *-* | *-* | *-* |
|  | **ID9** | *Irritation, frustration* | *Frustration* | *Irritation and helplessness* |
| **PHYSIOLOGICAL REACTIONS** | **ID1** | *-* | *-* | *-* |
|  | **ID3** | *-* | *Relaxed* | *-* |
|  | **ID6** | *-* | *-* | *-* |
|  | **ID7** | *-* | *-* | *.* |
|  | **ID9** | *Tension, agitation* | *Tension, agitation and restless* | *Headache, stomach pain* |
| **CRAVING (MAX)** | **ID1** | *0* | *0* | *0* |
|  | **ID3** | *0* | *2* | *3* |
|  | **ID6** | *2* | *2* | *0* |
|  | **ID7** | *0* | *10* | *10* |
|  | **ID9** | *9* | *10* | *3* |
